# Musical Scales in Tone Sequences Improve Temporal Accuracy

**DOI:** 10.3389/fpsyg.2018.00105

**Published:** 2018-02-06

**Authors:** Min S. Li, Massimiliano Di Luca

**Affiliations:** Centre for Computational Neuroscience and Cognitive Robotics, School of Psychology, University of Birmingham, Birmingham, United Kingdom

**Keywords:** tone frequency, expectation, perceived timing, temporal sensitivity, musical scale, isochrony

## Abstract

Predicting the time of stimulus onset is a key component in perception. Previous investigations of perceived timing have focused on the effect of stimulus properties such as rhythm and temporal irregularity, but the influence of non-temporal properties and their role in predicting stimulus timing has not been exhaustively considered. The present study aims to understand how a non-temporal pattern in a sequence of regularly timed stimuli could improve or bias the detection of temporal deviations. We presented interspersed sequences of 3, 4, 5, and 6 auditory tones where only the timing of the last stimulus could slightly deviate from isochrony. Participants reported whether the last tone was ‘earlier’ or ‘later’ relative to the expected regular timing. In two conditions, the tones composing the sequence were either organized into musical scales or they were random tones. In one experiment, all sequences ended with the same tone; in the other experiment, each sequence ended with a different tone. Results indicate higher discriminability of anisochrony with musical scales and with longer sequences, irrespective of the knowledge of the final tone. Such an outcome suggests that the predictability of non-temporal properties, as enabled by the musical scale pattern, can be a factor in determining the sensitivity of time judgments.

## Introduction

Perceived timing does not necessarily represent the objective time (e.g., Woodrow, 1935; Allan, 1979), as perception can be influenced by stimulus repetitions, sequence patterns, and expectations (e.g., Jones, 1976; Hirsh et al., 1990; Rose and Summers, 1995). The simplest form of temporal pattern, the repeated presentation of identical stimuli separated by identical intervals, has been shown to lead to increased temporal sensitivity in detecting anisochrony, and such an increase has been quantitatively captured by several models (Schulze, 1978; Drake and Botte, 1993; Ivry and Hazeltine, 1995; Miller and McAuley, 2005; ten Hoopen et al., 2011; Li et al., 2016). Similar perceptual influences of patterns on the precision of discrimination performance occur also when the pattern is more complex (Barnes and Jones, 2000; McAuley and Jones, 2003). Such improvements in temporal sensitivity have been interpreted in several ways, including an averaging process for the perceptual representation of interval durations (Schulze, 1978), the effect of sensory predictions generated by expectations and conditional probability of future event (Nobre et al., 2007), or the influence of neuronal oscillation-based predictive timing (Arnal and Giraud, 2012) which relates to the idea that rhythmic sequences entrain low-frequency neural oscillations enhancing sensory processing of in-phase stimuli (e.g., Jones, 1976; Lakatos et al., 2008; Ng et al., 2012; Cravo et al., 2013; Horr et al., 2016). So far, a large portion of the literature highlighted the perceptual benefits of temporal patterns in temporal judgments, but there has been also an interest in investigating how non-temporal patterns affect discrimination of time (e.g., Micheyl et al., 1998; Jones, 2009; Okazaki and Ichikawa, 2016). In this work, we will analyze whether some of these accounts based on the presence of temporal patterns, hold for sequences with non-temporal patterns.

From as early as the 19th century, numerous studies proposed a close relationship between pitch, melody and time (Stumpf, 1890; Divenyi and Danner, 1977; Long, 1977; Hébert and Peretz, 1997) that stem from two observations suggesting that temporal and tonal properties find a connection point in music. Firstly, the auditory modality has been traditionally recognized to have the highest temporal resolution of all senses (Hirsh and Sherrick, 1961), i.e., temporal attributes are most precise in human hearing than in any other sense. Thus, audition seemed to be the modality best tailored to process temporal properties, whether they are the frequency of a tone or its timing in a melody. Secondly, rhythm in music provides temporal cues which not only lead to temporal expectancies in complex melodic phases, but such temporal cues become a fundamental element in the perception of the musical piece (Cariani, 1999; Large and Palmer, 2002). There are several examples of the scientific investigation in support of the connection between temporal and tonal perception. For instance, König (1957) demonstrated a worse pitch discrimination with longer intervals (up to 5 s). Temporal regularity has been shown to help with implicit pitch structure learning (Jones et al., 2002; Selchenkova et al., 2014). Recent research, (for example, Kinney and Forsythe, 2012) showed positive influences of melody on various timing tasks, such as interval reproductions. In summary, the literature hints at an association between time and music where tone discrimination is facilitated or hindered by temporal properties of the stimulus. But because the results have been obtained with a range of different methods, it is difficult to infer whether the opposite is true, i.e., to what degree the structure of the sequence in the tonal domain can influence sensitivity to temporal properties, like the detection of deviations from a rhythm.

Other than the precision with which temporal judgments can be performed, recent studies have also been concerned with the presence of biases in the discrimination of temporal properties in sequences of stimuli. The perception of intervals in an isochronous sequence of identical stimuli is affected by a bias, as sequences need to be accelerated to be perceived to be isochronous (Di Luca and Rhodes, 2016). In accordance to this tendency to perceive accelerating sequences as being isochronous and consequently produce sequences that naturally speed up (Spence and Parise, 2010; Wackermann et al., 2014), it has been found that the last interval in a sequence appears shorter than it should, with an effect consistent with a perceptual acceleration of the last stimulus (Di Luca and Rhodes, 2016; Li et al., 2016). The presence of such biases in the perception of temporal properties is, as in the case of precision just discussed, affected by whether sequences have an organization, like the one music can provide. It has been shown, for example, that perceived duration largely depends on the stimulus context and on the events that occur during that particular duration of time (Ornstein, 1975; Block, 1978; Horr and Di Luca, 2015b). Similarly, Clynes and Walker (1986) found that musical concepts influenced the stability and accuracy of timing in musical performances. Despite the suggestion that temporal judgment and motor behavior in time are two distinct sensory attributes (London, 2011), the perception of musical time has also been shown to be biased by musical characteristics (Longuet-Higgins and Lee, 1982; Grisey, 1987). For instance, Boltz (1989) found the musical endings affected duration judgments, so that an unexpected tonic ending made the last interval to appear shorter compared to cases where the music finished with an expected tone. Such tendency is similar to what found by Horr and Di Luca (2015a), who showed that temporal regularity significantly increased the perceived duration of intervals compared to irregular ones. Interestingly for this paper, changing the regularity in tone frequency did not bias perceived duration. Clarke and Krumhansl (1990), instead, failed to identify a relationship between the perceived duration of a brief passage of music and the variety of the musical structure. Such a lack of an influence can be attributed to the recruitment of a group of trained musical experts to take part in the experiments. Due to the inconsistent use of experimental tasks such as perceptual judgments, sensorimotor synchronization and musical performances, as well as the difference in the populations tested in previous literature (i.e., musicians and non-musicians), it is difficult to draw a clear picture of the relation between sequence structure and bias in subjective timing. In particular it is not clear how to interpret the difference in perceived timing between regular and irregular sequences, which could be due to a decrease in otherwise biased timing with a structure or, on the contrary, an unexpected property of a stimulus is the culprit in creating temporal biases. Here we attempt to answer this question by studying how a non-temporal structure affects the accuracy in perceived timing.

To look into the effect of tones patterns on the precision of temporal discriminability and perceived timing, we cannot rely on duration reproduction tasks (Kinney and Forsythe, 2012) because motor variability has the effect of decreasing measurement precision. Instead, we will employ a temporal discrimination task that uses the two-alternative forced choice (2AFC) ‘early or late’ judgment (see Li et al., 2016). To estimate changes in temporal sensitivity (Hansen and Pearce, 2014) and biases in perceived timing, we will employ respectively the Just Noticeable Difference (JND) and the Point of Subjective Equality (PSE) calculated from each of the participants’ distribution of ‘late’ responses. Our experiment intentionally avoided recruiting musicians, as it has been shown that they obtain higher levels of performance in behavioral tasks (Repp and Doggett, 2007; Petrini et al., 2009; Matthews et al., 2016) do not exhibit perceptual biases (Clarke and Krumhansl, 1990) that can be explained by different cortical connectivity compared to the normal population (Lee and Noppeney, 2011).

In addition, to specifically avoid the confounding factors deriving from rhythm and melody, we analyze the interaction between sequence type and temporal structure using one of the simplest forms of tonal structure. That is, we test whether the arrangement of tones in a musical scale or in a sequence of random tones influences the detection of deviations from isochrony. Knowing whether there is an influence will contribute to the understanding of predictions and expectations within a sequence of stimuli on perception, as suggested by recent computational accounts (i.e., Jazayeri and Shadlen, 2010; Di Luca and Rhodes, 2016; Shi and Burr, 2016). We will also study whether knowing which tone is the one to be judged is sufficient to increase precision, i.e., by allowing participants to expect the tone and allocate the appropriate attentional resource. To do this, in Experiment 1, the final tone will vary across trials, whereas in Experiment 2, the final tone will always be presented with the same pitch (note A; 440 Hz).

## Materials and Methods

### Participants

A total of 42 non-musician undergraduate students (35 females, 19.6 ± 2.4 years), with self-reported normal hearing were recruited by the Research Participation Scheme of University of Birmingham. Participants were divided into two groups that took part either in Experiment 1 or in Experiment 2. They gave informed consent before taking part and were rewarded with either course credits or a payment of 6GBP/h. Ethical guidelines of the Declaration of Helsinki have been followed and were approved by the Science, Technology, Engineering and Mathematics (STEM) Ethics Committee of the University of Birmingham.

### Experimental Design

Participants were presented via Soundlab/Electrovision A069 Mono Earpiece headphones (with cup clip) with 3, 4, 5, or 6 60 ms tones, spaced 700 ms apart, except for the final tone whose time could deviate by 0, ±20, ±40, ±60, ±80, ±100, ±150, ±200 ms. The length of a trial ranged from 1380 to 4060 ms depending on the length of the sequence and the anisochrony of the final tone. The four sequence lengths were randomly intermixed in a block. At the end of each trial, participants pressed one of two keys to indicate whether the final tone in the sequence was ‘early’ or ‘late’ compared to the expected regular timing. Participants were offered the possibility to take a break at three points during the experiment.

All trial types resulting from the combination of sequence type (2 values: random and scale), sequence lengths (4 values), and anisochronies of the final tone (15 values) were repeated 8 times at random resulting in 960 trials per participant. We analyzed the proportion of ‘late’ responses at each anisochrony of the final tone, to obtain a distribution for each sequence length and sequence type. The Spearman-Kärber method (Ulrich and Miller, 2004) was employed to analyze the data, where the PSE was obtained by calculating the first order moment of the monotonized difference (Klein, 2001) between successive proportions of responses, while the JND was obtained by calculating the second order moment. The *post-hoc* tests were conducted with the JND and PSE values obtained to confirm the differences between each condition tested.

#### Experiment 1

**FIGURE 1:**
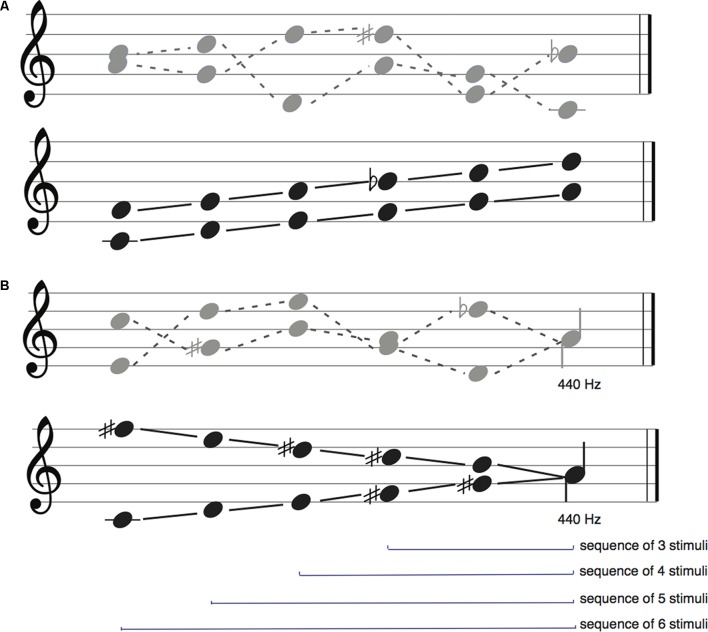
**(A)** Sequence type conditions in Experiment 1. The top row in gray depicts two examples of random sequences, while the bottom row in black depicts two of the four scale sequences. **(B)** Sequence type conditions in Experiment 2 where sequences always ended with the same tone. The top row depicts two examples of random sequences, while the bottom row in black depicts two of the four scale sequences.

For the scale condition, the sequence of tones was one of the four ascending diatonic scales: F major, C major, D major and E major, with tone frequencies ranging from 261.63 to 587.3 Hz. For the random condition, to avoid any sort of tonality, the frequency of each tone was randomly selected from the range of those employed in the scale condition. The final tones of the sequence were varied in all trials (non-fixed final tone condition). See **Figure 1A**.

#### Experiment 2

For the scale condition, the sequence of tones was one of the four ascending and descending diatonic scales: E Major, C major, E minor, C minor, ranging from 261.63 to 740 Hz. For the random condition, the note was randomly selected. To control the predictability in this particular experiment, the final tone of sequences was always 440 Hz (fixed final tone condition). See **Figure 1B**.

## General Results

**FIGURE 2:**
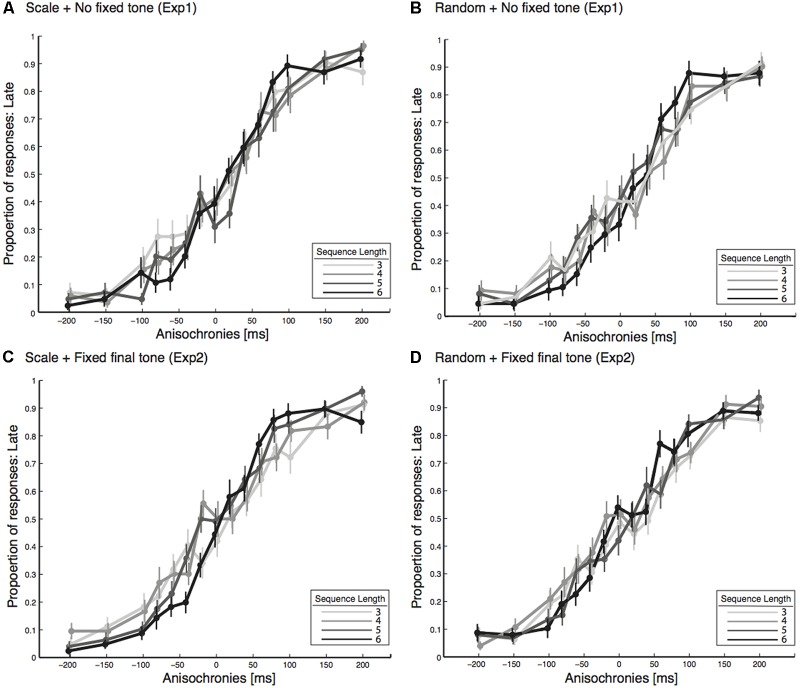
The proportion of “late” responses as a function of anisochrony of the final tone in the two experiments. Such data were analyzed using the Spearman–Kärber method to derive the PSE and JND values shown in **Figures 3**, **4**. **(A)** Shows the scale condition in Experiment 1, which had non-fixed final tone. **(B)** Shows the random condition in Experiment 1, which had non-fixed final tone. **(C)** Shows the scale condition in Experiment 2, which had the final tones always fixed at 440 Hz. **(D)** Shows the random condition in Experiment 2, which had the final tones always fixed at 440 Hz. All error bars represent the standard error of the mean.

**FIGURE 3:**
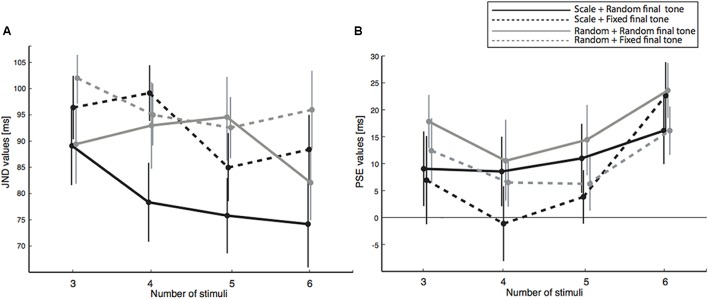
JND and PSE values obtained in the four conditions (two sequence types and two final tone fixing conditions) as a function of the four sequence lengths. **(A)** Shows the JND values of scale and random sequences with fixed and non-fixed final tone. **(B)** Shows the PSE values. Positive PSE values represent an equal proportion of “early” and “late” responses obtained with tone presented later than expected, which is consistent with an acceleration in the perceived timing of the final tone. All error bars represent the standard error of the mean.

**FIGURE 4:**
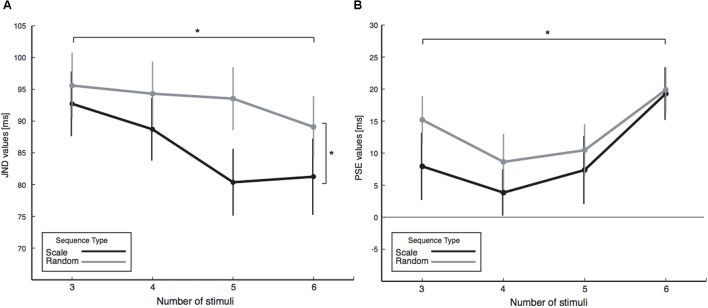
JND and PSE values as a function of sequence type and four sequence lengths, obtained by collating the values obtained from Experiment 1 and 2 (as we find no significant difference between them). **(A)** JND values evincing an improvement in performance due to sequence length and sequence type. **(B)** PSE values indicating that the final tone should be presented later than expected to be perceived isochronous, and the amount required increases with longer sequences. All error bars represent the standard error of the mean.

From the proportion of response data (**Figure 2**), we calculated PSE and JND values. JND values are shown in **Figure 3A** for each of the tested conditions. We conducted a three-way (one between and two within) ANOVA (and in parallel, a Bayesian mixed ANOVA) with the JND values, where the two final tone conditions (non-fixed, Experiment 1, and fixed at 440 Hz, Experiment 2) served as the between factor, two sequence types (random and scale) as the first within factor, and four sequence length (3, 4, 5, and 6 tones) as the second within factor. We did not find a significant difference in JND due to the final tone conditions [Experiment 1 vs. Experiment 2, *F*(1,40) = 1.4, *p* = 0.236, $\eta_{p}^{2}$ = 0.04, *BF_10_* = 0.62], as shown in **Figure 3A**. **Figure 4A** shows that sequence type changed JND by roughly 9% when compared random to scale sequences [*F*(1,40) = 10.5, *p* = 0.002, $\eta_{p}^{2}$ = 0.21, *BF_10_* = 27.8]. In addition, results demonstrated detectability to anisochrony changes with different sequence lengths [*F*(3,120) = 4.3, *p* = 0.007, *BF_10_* = 1.4], as shown in **Figure 4A**. No interaction was significant (all *p* > 0.2).

To gain further insight on the influence of sequence length on JNDs, we fitted a two-parameter regression line to the JND values of each participant, which shows a decrease of 3.2 ± 0.9 ms [single sample *t*-test of the slopes of the regression against 0, *t*(41) = -3.4, *p* = 0.001] and an intercept of 100.5 ± 5.3 ms.

We now turn our attention to the PSE values (**Figure 3B**). First, we conducted single sample *t*-test on PSE values against zero (presented in **Table 1**). The *t*-test results reported significant acceleration in perceived timing with the longest sequences (6 tones) for all four conditions. In addition, the PSE deviates from 0 for sequences composed of 3 and 5 tones in the non-fixed final tone condition only. Moreover, a regression line fitted to the PSE values and passing through the origin showed that the final tone needs to be presented 3.2 ± 0.4 ms more delayed to be perceived to be isochronous for each additional tone composing the sequence [*t*(41) = 5.4, *p* < 0.001].

**Table 1:** Single-sample Bonferroni corrected *t*-test on PSE values against zero.

	Sequence lengths		3	4	5	6
Experiment 1 (non-fixed final tone)	Scale	*t*(20) =	1.29	1.35	1.70	2.57
		*p* =	0.212	0.193	0.105	^∗^0.018
	Random	*t*(20) =	3.64	1.44	2.22	4.64
		*p* =	^∗^0.002	0.164	^∗^0.038	^∗^<0.001
Experiment 2 (fixed final tone)	Scale	*t*(20) =	0.85	-0.15	0.76	3.63
		*p* =	0.405	0.884	0.447	^∗^0.002
	Random	*t*(20) =	2.07	1.0	1.25	3.58
		*p* =	0.052	0.150	0.225	^∗^0.002

*Asterisks highlight p-values lower than 0.05*.

We assessed the influence of the experimental condition on PSE values with a three-way mixed ANOVA with the same factors used for the JND analysis. We did not observe a significant influence of the between factor final tone (fixed or non-fixed) on the PSE values [*F*(1,40) = 1.0, *p* = 0.334, $\eta_{p}^{2}$ = 0.02, *BF_10_* = 0.316]. The within factor sequence type (random or scale) also did not have an influence on PSEs [*F*(1,40) = 2.0, *p* = 0.169, $\eta_{p}^{2}$ = 0.05, *BF_10_* = 0.330]. In accordance with the results of the regression, we found that the timing at which the final tone was perceived to be isochronous changed across the different sequence lengths [*F*(3,120) = 4.4, *p* = 0.006, *BF_10_* = 5.67]. The effect of sequence length is best evidenced in **Figure 4B**, where we collapsed the non-influent final tone factor. No interaction between factors was present (all *p* > 0.5).

## Discussion

Our data replicated the improvement in sensitivity with longer sequences that have been reported previously (e.g., Schulze, 1978; ten Hoopen et al., 2011; Li et al., 2016). According to Schulze (1978), the reason for the performance change has been linked to the integration of multiple sensory estimates by a running average, which leads to a more precise and optimal representation. Recent accounts have framed the sensitivity improvement in terms of iterative interaction between sensory signals and temporal expectation, according to the rules of Bayesian inference (Di Luca and Rhodes, 2016). Such framework has been previously applied to explain several related phenomena in duration judgments. One of these was the regression to the mean interval when participants are presented with a range of durations during a block of trials (Jazayeri and Shadlen, 2010). Results were interpreted by advancing the hypothesis that a temporal prior and a cost function can account for the bias in the responses toward the mean and by showing how such a bias depended on the average duration of the range of intervals. Shi et al. (2013) proposed that an explanation based on the Bayesian Observer Model parallels information-processing ones, but such an explanation is normatively based on the reduction of temporal uncertainty. Our results are qualitatively consistent with an explanation based on such an uncertainty-reduction principle, and that the effect appears to be modulated by the pattern of non-temporal properties.

**FIGURE 5:**
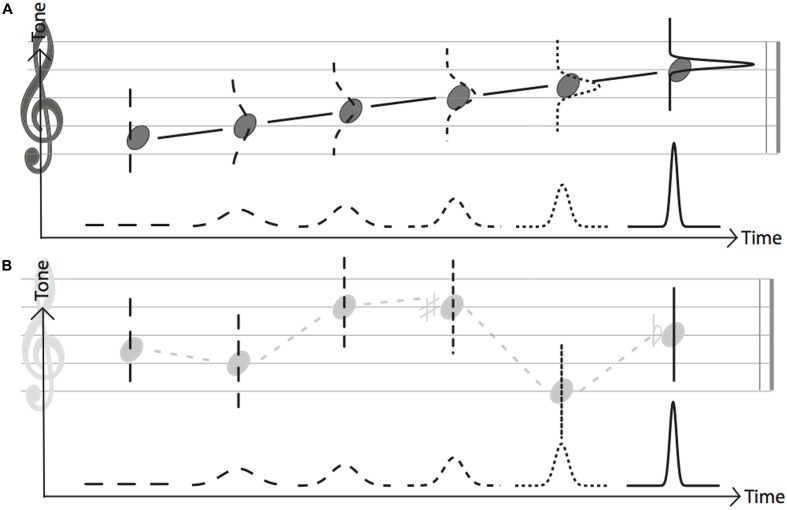
Graphic illustrating how predictability could influence temporal sensitivity. The distributions represent the predictability of time of upcoming tones at each position in an isochronous sequence, where a flat dashed line means no prediction and a narrow peak means great predictability. **(A)** Shows a progressive increase in predictability in the tone dimension and in the temporal dimension with an isochronous scale-toned sequence. **(B)** Shows an increased predictability in the time domain as the length of sequence increase, however, the random sequence does not lead to successive expectations of tone frequency, thus leading to a lower overall predictability of stimulus properties, leading to the lower precision in the discrimination of isochrony that we find here.

Our results indicate that temporal discrimination is more precise with sequences whose tones are arranged in a scale, rather than having tones arranged in a random sequence, and that this pattern is present irrespectively of the knowledge of the final tone. We speculate that equal temperament (scale-step) in the scale condition functioned as a ‘standard,’ which effectively generated musical expectation in the auditory sequence. Such scaled pattern arranged with simple tonality worked as a ‘physical attribute’ (Haluska, 2003) to a subjective perceptual experience. The influence of the regular scale-steps, as compared to atonality, led to the prediction of the frequency of the next tone and, in turn, to better coding of sensory information. Our data suggest that the sensory improvement was present not only in the pitch domain as demonstrated in the previous findings (i.e., Jones and Boltz, 1989; Selchenkova et al., 2014), but also in the time domain as shown by an increased temporal sensitivity. In addition to isochrony as shown in previous studies (i.e., Schulze, 1978), equal scale-steps in tonal structure also contribute to creating expectations, which allow more sensitive predictions of up-coming sensory events. We hypothesize that the process underlying such an improvement is akin to the formation of temporal priors which has been postulated in isochronous and equal pitch sequences (i.e., Di Luca and Rhodes, 2016). In the time domain, it has been advanced that the process is based on the projection of expectations for future stimuli according to the rules of Bayesian inference, which integrates *a priori* knowledge with sensory evidence (Jazayeri and Shadlen, 2010). If we extend the process to the tone domain, the presence of a scale allows the prediction of the frequency of the next tone, which combined with the prediction of the timing of the next tone, should iteratively improve the allocation of resources and the precision of sensory judgments (**Figure 5**). The current outcomes suggested a broader definition of ‘patterning’ in auditory perception, which should no longer be limited to the tone structure and temporal rhythms taken alone, but it should consider that the non-temporal characteristics can have an impact on temporal judgments (and vice versa). The findings denoted the direct influence of pattern in tonal structures on time perception.

In our experiment, in addition to precision, we investigated temporal accuracy by looking at the timing at which participants reported the final tone to appear to be isochronous. We find a general bias to report tones presented later than regular to be isochronous, replicating previous findings (Li et al., 2016). This result is consistent with a perceptual acceleration of stimuli presented in a sequence (Di Luca and Rhodes, 2016). The finding can be accounted for by an asymmetric representation of stimuli in the time domain (for details, see Di Luca and Rhodes, 2016). The manipulation of sequence length increases the anticipatory effect, but the tonal patterns seem to have no influence. Rather, the current PSE outcomes reported an anticipatory behavior in time, suggesting the influence of an attentional phenomenon consistent with *prior entry* (Titchener, 1908). Analogous to the effects of sensory entrainments (Rohenkohl and Nobre, 2011), it is a sensory characteristic of acceleration when a stimulust was attended to, the sensory processing was prioritized and facilitated. The phenomenon has been discussed and captured under various conditions, including the increased number of intervals in a sequence (for a review, see Spence and Parise, 2010; Li et al., 2016). In terms of the manipulation of various sequence lengths, longer sequences with more isochronic intervals usually generate more precise temporal expectations. As the number of tones in a sequence increases, the chances of a stimulus being temporally deviant also increased accordingly enhancing the attention deployed on the next possible timing of upcoming sensory events resulting in an anticipatory perception.

However, the fact that our PSE results did not highlight a different bias due to the tone structure does not appear to be in line with Dynamic Attending Theory proposed by Jones (1987) and Jones et al. (2002). Such proposal predicts instead that non-temporal patterns like music and tone scales, should lead to a modulation of attention and thus to a change in facilitation with consequent perceptual bias, that instead we did not find. A similar attempt to show sensory facilitation has been published recently, using the original pitch comparison task. Similar to our results, Bauer et al. (2015) showed that anticipatory behavior was also not present, possibly because in their stimuli the melody was ignored, and instead the temporal information was utilized for sensory expectation. In addition, they argued that a pitch comparison task may not be the most replicable and suitable judgment for investigating dynamic attending in audition. Here we showed that temporal judgments showed no evidence in the anticipatory attending as well. The failure of our and Bauer et al.’s 2015 attempts to find a bias in perceived timing could be also due to the simplicity of music in the sequences. In support of this possibility, we observe that Jones (1987) introduced several musical properties that were classified as dynamic elements that could facilitate the perceived onset of an auditory tone. This included the melodic accents, harmony and beat variations. The configuration of these dynamic characteristics in music were beyond simple patterns and followed a much more complex musical composition rule.

The current study exploited simple types of patterns in tones structure and timing to measure whether time perception is affected. We succeeded in showing an influence of a simple tonal pattern on temporal sensitivity, but such a difference is not associated with a change in bias. Moreover, we find a change in both precision and accuracy depending on sequence length. We explain such a pattern of results suggesting a predicting mechanism similar to the one hypothesized to regulate perception of regular temporal intervals. Here, in addition to temporal expectancies, tonal expectancies generated by a repetitive tone-gap can improve participants’ predictability of future sensory events. In sum, our results demonstrated the effectiveness of non-temporal patterns in the time dimension. Future research can consider manipulating harmony, chords, different auditory sources (i.e., vocal, instrumental stimuli), or signal reliability to further investigate how patterns influence the precision of temporal judgments. This type of research can be potentially combined with visual and tactile stimuli to provide a comprehensive understanding of musical and temporal perception.

## Author Contributions

ML and MDL contributed to the design and implementation of the research. ML carried out the experiment and wrote the manuscript with support from MDL who supervised the project.

## Conflict of Interest Statement

The authors declare that the research was conducted in the absence of any commercial or financial relationships that could be construed as a potential conflict of interest.
